# Hyperglycemia-associated Alzheimer’s-like symptoms and other behavioral effects attenuated by *Plumeria obtusa* L. Extract in alloxan-induced diabetic rats

**DOI:** 10.3389/fphar.2022.1077570

**Published:** 2022-12-16

**Authors:** Sumeera Naz, Imran Imran, Muhammad Asad Farooq, Syed Adil Hussain Shah, Iqra Ajmal, Zartash Zahra, Aqsa Aslam, Muhammad Irfan Sarwar, Jaffer Shah, Ambreen Aleem

**Affiliations:** ^1^ Department of Pharmacology, Faculty of Pharmacy, Bahauddin Zakariya University, Multan, Pakistan; ^2^ Shanghai Key Laboratory of Regulatory Biology, School of Life Sciences, East China Normal University, Shanghai, China; ^3^ Gujrat Institute of Management Sciences, Pir Mehr Ali Shah Arid Agriculture University Rawalpindi, Gujrat Campus, Gujrat, India; ^4^ Department of Health, New York, NY, United States

**Keywords:** Alzheimer, anxiolytic, anti-depressant, learning, memory, anti-diabetic

## Abstract

Diabetes mellitus is a chronic metabolic complaint with numerous short- and long-term complications that harm a person’s physical and psychological health. *Plumeria obtusa* L. is a traditional medicine used in the treatment of diabetes to reduce complications related to behavior. *Plumeria* is a genus with antipsychotic activities. The objective of this study was to examine the effects of a methanolic extract of *Plumeria obtusa* L. in the attenuation of diabetes, on symptoms of Alzheimer disease, and on other associated behavioral aspects. A single dose of alloxan was administered to an experimental group of rats to induce development of diabetes (150 mg/kg, intraperitoneal) and the rats were then administered selected doses of methanolic extract of *Plumeria obtusa* L. (Po.Cr) or glibenclamide (0.6 mg/kg) for 45 consecutive days. Behavioral effects were evaluated using three validated assays of anxiety-related behavior: the open field test, the light and dark test, and the elevated plus maze. Anti-depressant effects of *Plumeria obtusa* L. were evaluated using the forced swim test (FST) and memory and learning were assessed using the Morris water maze (MWM) task. Po.Cr was also evaluated for phytochemicals using total phenolic content (TPC), total flavonoid content (TFC), and high-performance liquid chromatography assays, and antioxidant capability was assessed through assays of DPPH radical scavenging, total oxidation capacity, and total reducing capacity. In the alloxan-induced model of diabetes, the administration of Po.Cr and glibenclamide for 45 days produced a marked decrease (*p* < 0.001) in hyperglycemia compared to control animals. Po.Cr treatment also resulted in improvement in indicators, such as body weight and lipid profile (*p* < 0.05), as well as restoration of normal levels of alanine transaminase (ALT) (*p* < 0.001), a biomarker of liver function. Diabetic rats presented more Alzheimer-like symptoms, with greater impairment of memory and learning, and increased anxiety and depression compared to non-diabetic normal rats, whereas treated diabetic rats showed significant improvements in memory and behavioral outcomes. These results demonstrate that Po.Cr reversed alloxan-induced hyperglycemia and ameliorated Alzheimer-related behavioral changes, which supports additional study and assessment of conventional use of the plant to treat diabetes and associated behavioral complications.

## 1 Introduction

Diabetes mellitus (DM) is a heterogeneous metabolic complaint involving increased blood glucose (Talha et al., 2022), which is a result of inadequate insulin secretion, diminished insulin sensitivity, or both. DM is a polygenic condition with increased reactive oxygen species (ROS) and basal metabolic rate, along with deficiency in lipoproteins and free radical scavengers, and impairment of organs due to oxidative stress (Shah and Khan, 2014; Behl et al., 2022). Additionally, complications of DM include psychiatric complaints such as depression and anxiety, neurodegenerative impairments, and cognitive decline (Ceretta et al., 2012a; Reus et al., 2016). Numerous studies have shown an association between the pathophysiology of DM and psychiatric disorders due to alterations in glucose metabolism, formation of ketone bodies, oxidative stress, and negative effects on neuroplasticity (Hassan et al., 2022a, Ceretta et al., 2012a). The harmful consequences of DM are worsened by oxidative stress and inflammation, which contribute to the induction of DM and its complications, play a crucial role in diabetic tissue damage, and are a major contributors to diabetic neuropathy (Ceretta et al., 2012b). Oxidative stress arises from the imbalance between ROS and antioxidant defensive mechanisms (Hassan et al., 2022b). Hyperglycemia can elevate the production of ROS and lead to the damage of numerous cellular components, such as proteins, nucleic acids, amino acids, and lipids (Gupta et al., 2017). Decreased levels of circulating antioxidants associated with diabetes may be one of the risk factors for Alzheimer disease and depression (Abduljawad et al., 2022; Kabra et al., 2022). DM is also associated with alterations in neurochemicals and hormones that can be linked with anxiety and depression. The co-morbidity of diabetes with anxiety disorders has demonstrated greater diabetic complications, greater pain, increased depression, and decreased quality of life (Smith et al., 2013). Diabetes and its complications can be reversed or prevented by effective control of blood glucose levels. The utilization of medicinal plants based on ancient practices has had a resurgence (Sarwar et al., 2011; Mahnashi et al., 2022; Zou et al., 2022), and plant-based compounds should be part of an advanced treatment strategy. Present-day medications for diabetes are hampered by limited effectiveness and adverse effects that range in seriousness from negligible weakness to death from severe hypoglycemia, hepatic and kidney damage, or chronic toxicity. These adverse effects of established treatments have led to replacement with alternative medicines and herbal products, as they are safe and cost-effective. The effective use of medicinal plants to treat diabetes and related complications has been established in experimental animal models.

*Plumeria obtusa* L., a member of the family Apocynaceae, is commonly known as white frangipani, chafa, and gul cheen. In the traditional system of medicines, leaves of *Plumeria obtusa* L. have been frequently used to treat hyperglycemia (Ali et al., 2014; Dogra, 2016; Mulaudzi et al., 2019; Bihani et al., 2021). Furthermore, it has been traditionally applied in the treatment of skin diseases, fever, pain, inflammation (Zhang et al., 2022), arthritis, and gastrointestinal ailments (Zhang et al., 2021), bacterial, fungal, and viral (esp. herpes zoster) infections, as well as in cancer treatment (Wong et al., 2011; Devprakash et al., 2012; Asiimwe et al., 2013; Shah et al., 2015; Lotankar et al., 2016). Decoction of leaves is commonly employed to treat wounds and skin infections, cerebral pain, and asthma, and as a laxative, antitoxin, or diuretic (Ali et al., 2014; Shah et al., 2015). Roots are applied for the treatment of asthma, constipation, dysentery, leprosy, ulcers, skin and liver maladies, and tumors. Previous pharmacological studies revealed the presence of antifungals, antimicrobials, and antivirals (Ali et al., 2014), as well as gastro-protective (Singh et al., 2012), laxative, diuretic, anti-tumor (Wong et al., 2011), and antioxidant activities in *Plumeria obtusa* L. (Dogra, 2016; Bihani et al., 2021). Some species of the genus *Plumeria* have been reported to have anxiolytic activities as well (Chatterjee et al., 2013). Phytochemical investigations of *P. obtusa* revealed the presence of tannins, triterpenoids, saponin, proteins, glycoside, flavonoids, essential oils, carbohydrates, and alkaloids (Singh et al., 2012). Furthermore, *Plumeria obtusa* L. (aerial parts) showed the presence of pentacyclic triterpenoids, including betulinic, oleanolic, and ursolic acids (Siddiqui et al., 1989; Devprakash et al., 2012; Alvarado et al., 2015). Another study reported that iridoids characterized as acetylplumieride coumarate and acetylplumieride-p-Z-coumarate are found in the plant, along with other constituents that include isoplumericin, plumieride, plumieride coumerate, and plumieride coumerate glucoside (Ali et al., 2014). Benzyl salicylate and benzyl benzoate are the essential oils found in *P. obtusa* (Devprakash et al., 2012).

*Plumeria obtusa* L. is a medicinally important plant with great potential and substantial traditional claims regarding its use to treat diabetes and associated Alzheimer-related and behavioral effects, but there remains a lack of sufficient experimental data to validate those claims. The purpose of this study was to examine the effect of the methanolic crude extract of *Plumeria obtusa* L. on the alloxan model of DM and associated Alzheimer-related and behavioral consequences, including cognitive impairment, anxiety, and depression.

## 2 Methods

### 2.1 Chemicals

Chemicals/reagents utilized in the experimental work were of analytical research grade. Methanol was procured from Duksan Pure Chemicals, Korea. Sodium chloride for normal saline was obtained from Otsuka, Pakistan, glucose from Merck, Germany, and alloxan and glibenclamide from Sigma-Aldrich, Germany.

### 2.2 Collection and pre-treatment of plant material

*Plumeria obtusa* L. (leaves) were collected from Multan in the spring of 2017. Sample “R.R. Stewart 565″” was preserved at Bahauddin Zakariya University (BZU), Institute of Pure and Applied Biology in Multan. Leaves were obtained, cleaned, dried, and ground into powder. A total of 1 kg of coarse powder was soaked in 80% v/v hydro-methanol for 7 days in an amber colored glass jar with occasional shaking. After filtering, the filtrate was dried in a rotary evaporator at 37°C and low pressure, and a viscous substance derived from the *Plumeria obtusa* (Po.Cr) leaves, with a yield of 12.6%, was produced. The extract was maintained at –20°C in an airtight, amber-colored vial for future experiments.

#### 2.2.1 Dosage preparation

Po.Cr was dissolved in 1 ml of normal saline and 0.1 g/ml of Po.Cr was delivered orally for all experiments.

### 2.3 Experimental animals and their care

Male Sprague-Dawley rats weighing between 150 and 260 g were used and were kept at the Faculty of Pharmacy’s animal house at BZU in Multan. Rats were kept in sawdust-lined polycarbonate cages with a 12 h light/dark cycle under regulated conditions. Rats were fed a high fat/carbohydrate-rich diet before induction of diabetes, and later fed regular rodent feed pellets containing 50% carbohydrates, 25% proteins, and 25% fats on a regular basis; the rats had free access to water.

### 2.4 Alloxan-induced experimental diabetes

On day 0 of the experiment, the selected rats were administered freshly prepared alloxan monohydrate (150 mg/kg/i.p.) in sterilized normal saline, after 12 h of fasting (Johar et al., 2018). Massive insulin discharge from the pancreas due to apoptosis of insulin producing beta cells generally leads to alloxan-induced hypoglycemia. Therefore, the rats were kept on 5% glucose for the following 24 h to prevent hypoglycemia.

### 2.5 Experimental design

The Po.Cr doses were selected based on preliminary experiments in our laboratory, in which rats were orally treated with four different doses: 100, 150, 250 and 500 mg/kg. For this study, rats were arbitrarily divided into five groups; details of grouping and dosing are given in Table 1. Drug and plant extract doses were administered to animals daily for 45 days *via* gavage feeding tube. After day 25, the animals were assessed using different behavioral tests, including the open field, light and dark, elevated-plus maze, forced swim test (FST), and Morris-water maze test. Body weight and blood glucose levels were assessed on alternate days using an electronic balance and glucometer, respectively.

**TABLE 1:** Layout of animal groups and treatment of the alloxan-induced diabetic rat model.

Layout of animal groups and treatment				
Group I	Group II	Group III	Group IV	Group V
Normal control	Alloxan	Alloxan + Glibenclamide	Alloxan + Po.Cr extract, p.o.	Alloxan + Po.Cr extract, p.o.
1 ml/kg N.S.	150 mg/kg	150 mg/kg + 0.6 mg/kg	150 mg/kg + 300 mg/kg	150 mg/kg + 500 mg/kg
Administered once daily *via* oral gavage from day 1 to day 45	Single dose i.p.	Administered once daily *via* oral gavage from day 1 to day 45		

On day 46, blood was taken from rats by cardiac puncture while under mild isoflurane (5% v/v) anesthesia (Kumar et al., 2017). Blood samples were immediately transferred to falcon tubes, kept at 15°C–25°C for an hour, and then centrifuged at 2,500 rpm for 15 min to obtain serum for biochemical analysis.

### 2.6 Behavioral tests

#### 2.6.1 Behavioral test for learning and memory

##### 2.6.1.1 Morris water maze test

A round water-filled swimming pool was utilized for this test, as previously described (Morris, 1984; Diegues et al., 2014). The apparatus comprised a large, dark water tank made of fiberglass, and was 150 cm in diameter, 50 cm in height, and full of water, at a temperature of 27 ± 1°C, to a depth of 30 cm. Non-toxic white dye was added to the water to make it opaque, and a platform of 29 cm in height and 10 cm × 10 cm in breadth was placed 1 cm below the surface of the water. The pool was separated into four equivalent quadrants, labeled northeast, southeast, southwest, and northwest. The platform was placed in the southwest quadrant and remained there throughout the experiment. On the higher border of the water pool, four indicators were set in the middle of the circumference of every quadrant. The position of the indicators was kept the same throughout the experiment. The apparatus was kept in the test room, with indirect light and a fixed video camera (Logitech, Webcam HD) on the ceiling to track the movement of the animals. The results were assessed using video capture and tracking via ANY-maze software. The animals were allowed to move freely and locate the platform only by means of distant signs placed in the experimental room. The time it took each rat to find and get on the platform was recorded. If the rat remained unable to find the platform within 90 s, it was put on the platform and left there for 30 s. The animal was then returned to its cage for 20 s prior to start of the next trial. The process was repeated by starting from another position in the pool according to the previous trial. Four consecutive trials were performed with each of the animals in similar order. The time to find the platform, i.e., escape latency, was measured during each trial.

On the fifth day, the platform was removed from the pool and a probe trial was performed. The animals were positioned in the pool opposite the prior platform-containing quadrant. The session lasted 90 s, during which the time spent in the targeted quadrant was noted.

#### 2.6.2 Behavioral tests of anxiety

##### 2.6.2.1 Open field test

The open field test (OFT) is a standard test used to assess the effects of test compounds on probing behavior and anxiety. The apparatus consisted of a square box with dimensions of 80 cm × 80 cm × 40 cm and made of white polyacrylic plastic. The apparatus was placed in the middle of the experiment room, which was properly illuminated and soundproof. At the beginning of the experiment, each rat was gently positioned in the center of the box and permitted to move freely for 5 min. The activity of each rat was recorded using a video camera and then analyzed *via* ANY-maze software version 5.3. The ANY-maze video tracking system facilitated analysis of behavioral experiments based on parameters such as total distance traveled and number of entries into the center zone or corner zone, including data on duration in the respective zones. Higher total number of entries and greater time spent in the central area are indicators of reduced anxiety (Turner and Burne, 2014).

##### 2.6.2.2 Light and dark aversion test

The light and dark box (L/D) was also used to examine the anxiolytic effects (Turner and Burne, 2014) of Po.Cr. The apparatus was made up of two plastic boxes with dimensions of 40 cm × 25 cm × 20 cm; one white and the other black. The two boxes were connected *via* a small opening of 7 cm × 7 cm. The animal was allowed to move freely from one box to the other through the opening. The apparatus was set on a clapboard of transparent plastic-covered wood. The transparent white box was brightly illuminated with a 60 W bulb located above the box.

After an hour of pretreatment with extract, each animal was positioned in the center of the white box facing the open hole and permitted to explore the apparatus for 5 min. The apparatus was cleaned using 70% IPA after every trial. The activity of each rat was recorded using a video camera and behavior was assessed using parameters that included number of entries and total time spent in the light and dark boxes (Doukkali et al., 2015; Manikkoth et al., 2016). Decreased activity of animals in the light compartment of a light/dark box indicates anxiety-like behavior (Castillo-Gomez et al., 2015).

##### 2.6.2.3 Elevated plus maze

For additional evaluation of anxiolytic effects of Po.Cr, the elevated plus maze (EPM), first developed by Lister in 1987, was employed (Adeyemi et al., 2010). This method was used with slight modification. The EPM apparatus was made of wood and the maze floor was made of black plexiglass. The maze consisted of two open arms of 110 cm in length and two opposite closed arms of 110 cm in length, with a 35 cm high wall that formed a plus sign and a central square of 10 cm × 10 cm. The entire apparatus was raised from the ground by approximately 50 cm. The apparatus was brightly illuminated by the lights in the experiment room. After an hour of treatment with extract, the rats were positioned in the middle of the apparatus facing towards one of open arms and the experiment was performed for 5 min. The apparatus was cleaned after every trial using 70% IPA. All trials were recorded using a video camera and behavior was assessed using the following parameters: number of entries into the open and closed arms, and total time spent in the open and closed arms (Tang et al., 2015). Increased open arm entries and time spent in the open arm are indicators of reduced anxiety.

#### 2.6.3 Behavioral test for depression

The forced swim test for evaluating the activity of anti-depressants was first used by Porsolt et al. (1977). In our study, the apparatus was made of a plexiglass cylinder (23 cm in diameter and 35 cm in height) that was filled with water (temperature 24°C–26°C). Each rat was placed in the water briefly as a test to ensure that it did not escape the container and that its feet did not touch the floor of the vessel (Tang et al., 2015). An hour after the preliminary test, the rat was subjected to FST and required to swim for 5 min. After completing the test, the rat was removed from the cylinder, dried with a towel, and placed under a heating fan for 15 min before being returned to its cage. After each trial, the water was removed from the cylinder and replaced with fresh water. The experiment was carried out in a brightly illuminated room. The activity of the animals was recorded using a video camera and then analyzed via ANY-maze software version 5.3. The following behavioral parameters were analyzed:

• Total time immobile (in seconds)
• Total time mobile (in seconds)

### 2.7 Phytochemical study

#### 2.7.1 Evaluation of total phenolic content

Folin-Ciocalteu reagent analysis (Fatima et al., 2015) confirms the presence of phenolic compounds in tested substances. Each well of a 96-well plate included 20 μl of a 4 mg/ml solution of Po.Cr in DMSO plus 90 μl of Folin-Ciocalteu reagent. When the initial 5 min incubation period was complete, 90 μl of Na_2_CO_3_ was added to the reaction. The absorbance of each reaction mixture was measured at 630 nm in an ELX800 microplate reader (BioTek, United States), using gallic acid (GA) as the standard. The study was repeated three times, and the results are expressed as mg gallic acid equivalents per gram of sample in dry weight (GAE/g DW).

#### 2.7.2 Evaluation of total flavonoid content

The flavonoid content was determined using a modified version of the aluminum chloride colorimetric technique (Fatima et al., 2015). Aluminum chloride solution (10%), potassium acetate (1.0 M), and distilled water (160 μl) were added to a plate containing 20 μl Po.Cr. After 30 min of incubation, absorbance of the reaction mixture was measured at 415 nm using a microplate reader. The flavonoid content was determined by repeating the experiment three times, and the results were expressed as mg of quercetin equivalents per gram of sample in dry weight (QE/g DW).

#### 2.7.3 HPLC analysis

HPLC was performed in accordance with previously published methods (Fatima et al., 2015), with slight modification through use of a binary gradient pump from the Agilent ChemStation Rev series 260 and 1,200 attached to a diode array detector. Solvents used as the mobile phase were labeled solvent A and solvent B. Solvent A contained methanol (10): acetonitrile (5): water (85): acetic acid (1) and solvent B contained methanol (60): acetonitrile (40): acetic acid (1). The flow rate was maintained at 1 ml/min. Stock solutions of numerous standards were prepared in methanol and sequentially diluted to the final concentrations of 10, 20, 50, 100, and 200 μg/ml. The absorption of Po.Cr was recorded at various wavelengths, including 257 nm for rutin, 279 nm for gallic acid and catechin, 325 nm for caffeic acid and apigenin, and 368 nm for myricetin, quercetin, and kaempferol, and the analysis was performed three times. For the detection of compounds, retention time and absorption spectra were compared with known standards.

### 2.8 *In vitro* assessment of antioxidant markers

#### 2.8.1 DPPH radical scavenging assay

The antioxidant capacity of Po.Cr was measured by its ability to scavenge the free radical 2,2-diphenyl-1-picrylhydrazyl (DPPH), with ascorbic acid acting as a reference standard (Fatima et al., 2015). The percent radical scavenging activity (RSA) and IC_50_ values were determined by spectrophotometric analysis. In 96-well plates, 180 μl of DPPH solution (9.2 mg/100 ml in methanol) were combined with four dilutions of Po.Cr (20 μl) to obtain concentrations of 200.0, 66.66, 22.22, and 7.406 μg/ml. The experiment was run, in triplicate, for 30 min at 37°C, and the absorbance was measured at 517 nm using a microplate reader. The percentage of resource savings due to scavenging was determined by:

$${\%\text{RSA}=\text{Ab}}_{c}{-\text{Ab}}_{s}{\;/\text{Ab}}_{c}*\;100$$

Ab_s_ = absorbance of sample (Po.Cr); Ab_c_ = absorbance of negative control.

#### 2.8.2 Estimation of antioxidant potential

To determine the antioxidant potential of Po.Cr, the phosphomolybdenum assay was used. A 0.1 ml aliquot of Po.Cr (4 mg/ml of DMSO) was combined with 0.1 ml of ascorbic acid (4 mg/ml) in 1 ml of reagent containing 0.6 M sulfuric acid, 28 mM sodium phosphate, and 4 mM ammonium molybdate. A blank solution was added to the same amount of solvent as done with the experimental sample. After 90 min in a boiling water bath at 95°C, the test tubes were cooled to room temperature. Using a PDA spectrophotometer (8354 Agilent Technologies, Germany), we compared the sample’s absorbance to that of the blank at 695 nm. When describing the antioxidant activity, the unit of measure used was milligrams of ascorbic acid equivalents (AAE) per gram of dry weight (Fatima et al., 2015).

#### 2.8.3 Total reducing power assessment

The reducing power of Po.Cr was evaluated using the potassium ferricyanide colorimetric assay, as previously published (Fatima et al., 2015). In short, a 200 μl aliquot of 4 mg/ml Po.Cr in DMSO was dissolved in 400 μl of 0.2 mol/L phosphate buffer and 1% potassium ferricyanide. The reaction mixture was incubated at 50°C for 20 min. The mixture was then centrifuged at 3,000 rpm in a solution of trichloroacetic acid (400 µl). A 100 µl aliquot of 0.1% FeCl_3_ and 500 µl of distilled water were added to the top layer. The absorbance at 700 nm was noted; an increase in absorbance of the reaction mixture indicated increased reducing power. The blank consisted of the above-mentioned reaction mixture plus 200 μl DMSO instead of the extract. The reducing power was articulated as mg AAE/g DW, and the assay was run in triplicate.

### 2.9 Statistical analysis

The behavioral test data were analyzed using two-way ANOVA and subsequent multiple Dunnett’s tests in GraphPad Prism (version 8.0.1), while the remaining experimental data were analyzed using one-way ANOVA and a subsequent Dunnett’s test. The results are reported as mean ± standard deviation; *p* < 0.05 indicates a statistically significant difference between groups.

## 3 Results

### 3.1 Po.Cr exhibits *in vivo* antidiabetic activity

**FIGURE 1:**
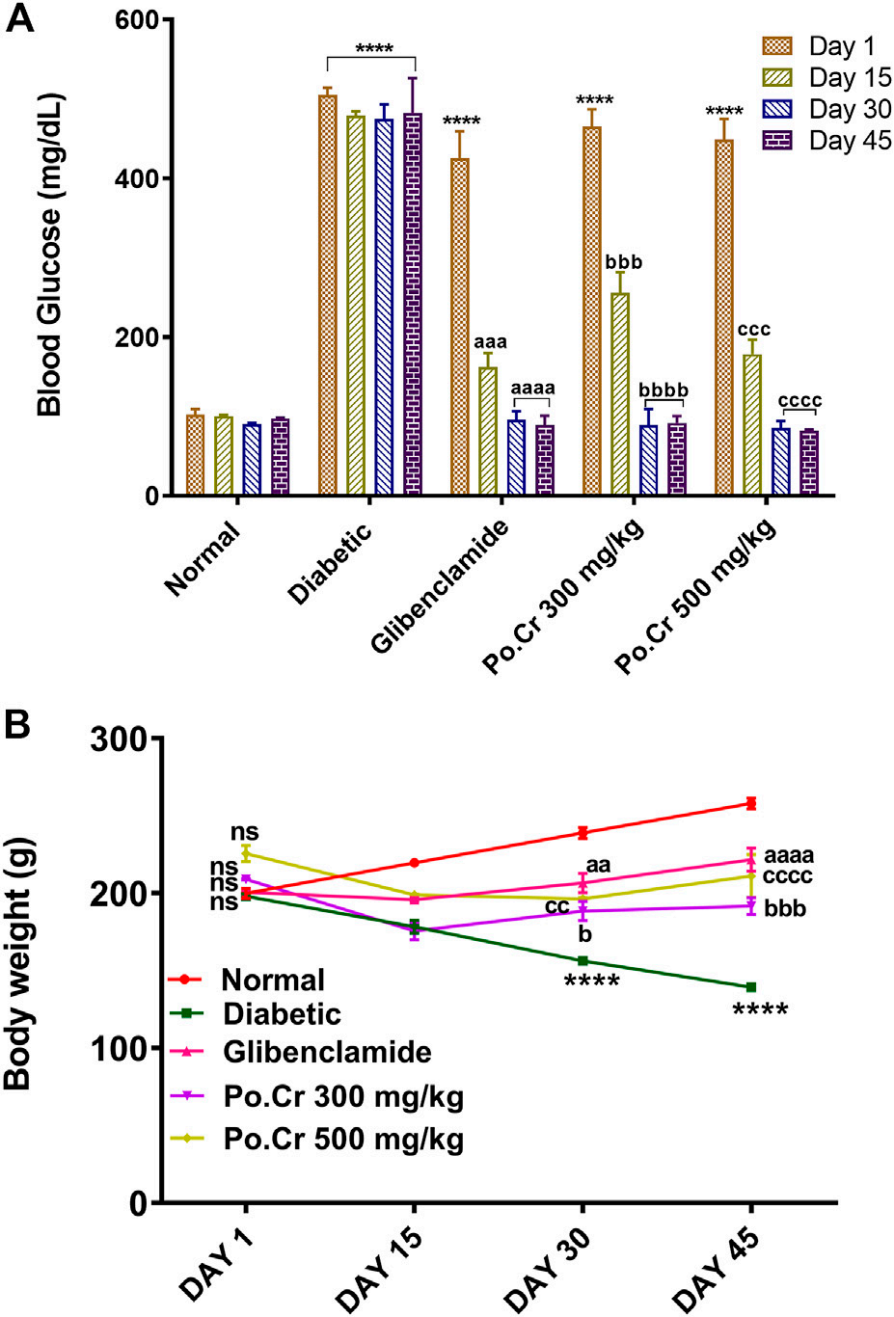
Graphical representation of the effects of methanolic extract of *Plumeria obtusa* L. on **(A)** blood glucose levels (mg/dl) and **(B)** body weight of rats. Group II (diabetic control) was compared to Group I (normal control), whereas all the treated groups (Groups III [glibenclamide] to V [Po.Cr]) were compared with Group II. ANOVA (two-way) and the multiple comparison Dunnett’s test were applied, and the data values are mean ± SEM. *p < 0.05 shows comparison of the diabetic control (Group II) to the normal control (Group I), whereas ^a^ *p* < 0.05, ^*b*^ *p* < 0.05, and ^c^ *p* < 0.05 indicate comparison of glibenclamide (Group III); Po.Cr, 300 mg/kg (Group IV); and Po.Cr, 500 mg/kg (Group V) *versus* the diabetic control group, respectively.

The blood glucose level remained higher in alloxan-induced diabetic rats compared to normal rats during the 45 days of the study (*p* < 0.001) (Figure 1A). Furthermore, rats treated with glibenclamide (Group III) and Po.Cr at the selected doses had significantly reduced blood glucose levels during the study compared to untreated rats (*p* < 0.001). The study revealed that Po.Cr had significant anti-diabetic activity in treated diabetic rats compared to untreated diabetic rats.

During the experimental period (45 days), untreated diabetic rats showed prominent weight loss, from 199.66 ± 7.19 to 139.67 ± 3.41 g, compared with normal healthy rats that showed weight gain, from 199.33 ± 5.73 to 258.6 ± 3.25 g. However, treatment with 300 or 500 mg/kg of Po.Cr protected diabetic rats from the significant weight loss observed in untreated diabetic rats (Figure 1B).

### 3.2 Po.Cr improves metabolic fitness of diabetic rats

Apart from alterations in glucose metabolism, DM is often associated with alterations in cholesterol metabolism and hepatic dysfunction (Aleissa et al., 2020). After administration of alloxan (150 mg/kg) to induce diabetes, the rats showed a noticeable increase in levels of total cholesterol (TC), triglycerides (TG), LDL cholesterol, and the liver function biomarker ALT, along with a reduction in HDL cholesterol levels in comparison to untreated controls (Group I). As expected, the standard diabetes drug glibenclamide (0.6 mg/kg) significantly reduced TC, TG, and LDL levels, reduced liver serum markers (*p* < 0.001), and increased HDL levels when compared to the diabetic group that did not receive glibenclamide (*p* < 0.05) (Table 2). Po.Cr significantly reduced harmful cholesterol biomarkers, including TC, TG, and LDL and led to an increase in ALT and HDL levels. Our results in Table 2 demonstrate that Po.Cr treatment can significantly improve the metabolic profile of diabetic rats.

**TABLE 2:** The levels of TC, TG, LDL, HDL, and ALT in blood serum (*n* = 8). Group II (diabetic control) was compared to Group I (normal control), whereas all the treated groups (Groups III [standard treatment] to V [Po.Cr]) were compared with Group II (diabetic control). ANOVA (two-way) and the multiple comparison Dunnett’s test were applied, and the data values are mean ± SEM. *p < 0.05 and ^a^ *p* < 0.05 indicate comparison of the diabetic control (Group II) to the normal control (Group I) and glibenclamide (Group III), respectively. Whereas ^b^ *p* < 0.05 and ^c^ *p*<0.05 indicate comparison of Po.Cr, 300 mg/kg (Group IV); and Po.Cr, 500 mg/kg (Group V) *versus* the diabetic control group, respectively.

Groups	Total cholesterol (mg/dl)	Triglycerides (mg/dl)	LDL (mg/dl)	HDL (mg/dl)	ALT (U/L)
Normal	90.45 ± 1.73	77.83 ± 1.39	22.16 ± 0.87	43.16 ± 1.01	28.83 ± 2.40
Diabetic	254.5 ± 1.67****	197.16 ± 1.09****	92.54 ± 1.79****	19.34 ± 1.75****	92.33 ± 3.34****
Glibenclamide 0.6 mg/kg	116.5 ± 3.74^aaaa^	124.23 ± 2.25^aaaa^	39.65 ± 0.73^aaaa^	40.3 ± 0.76^aaaa^	38.83 ± 1.83^aaaa^
Po.Cr 300 mg/kg	163.8 ± 1.88^bbb^	149.5 ± 1.87^bbb^	56.6 ± 2.04^bbb^	29.5 ± 0.51^bbb^	50.66 ± 1.86^bb^
Po.Cr 500 mg/kg	127.33 ± 1.31^cccc^	136.3 ± 1.79^cccc^	42.21 ± 1.42^cccc^	37.65 ± 0.88^cccc^	45.16 ± 2.17^cccc^

**** *p* < 0.001 and aaaa *p* < 0.001 show respective comparisons of diabetic control (Group II) to normal control (Group I) and glibenclamide (Group III). While, bbb *p* < 0.005 and cccc *p* < 0.001 show comparison of Po.Cr; 300 mg/kg (Group IV) and Po.Cr; 500 mg/kg (Group V) versus diabetic control group respectively.

### 3.3 Po.Cr decreases Alzheimer-like complications of diabetes and improves behavioral outcomes in diabetic rats

#### 3.3.1 Behavioral test for learning and memory

**FIGURE 2:**
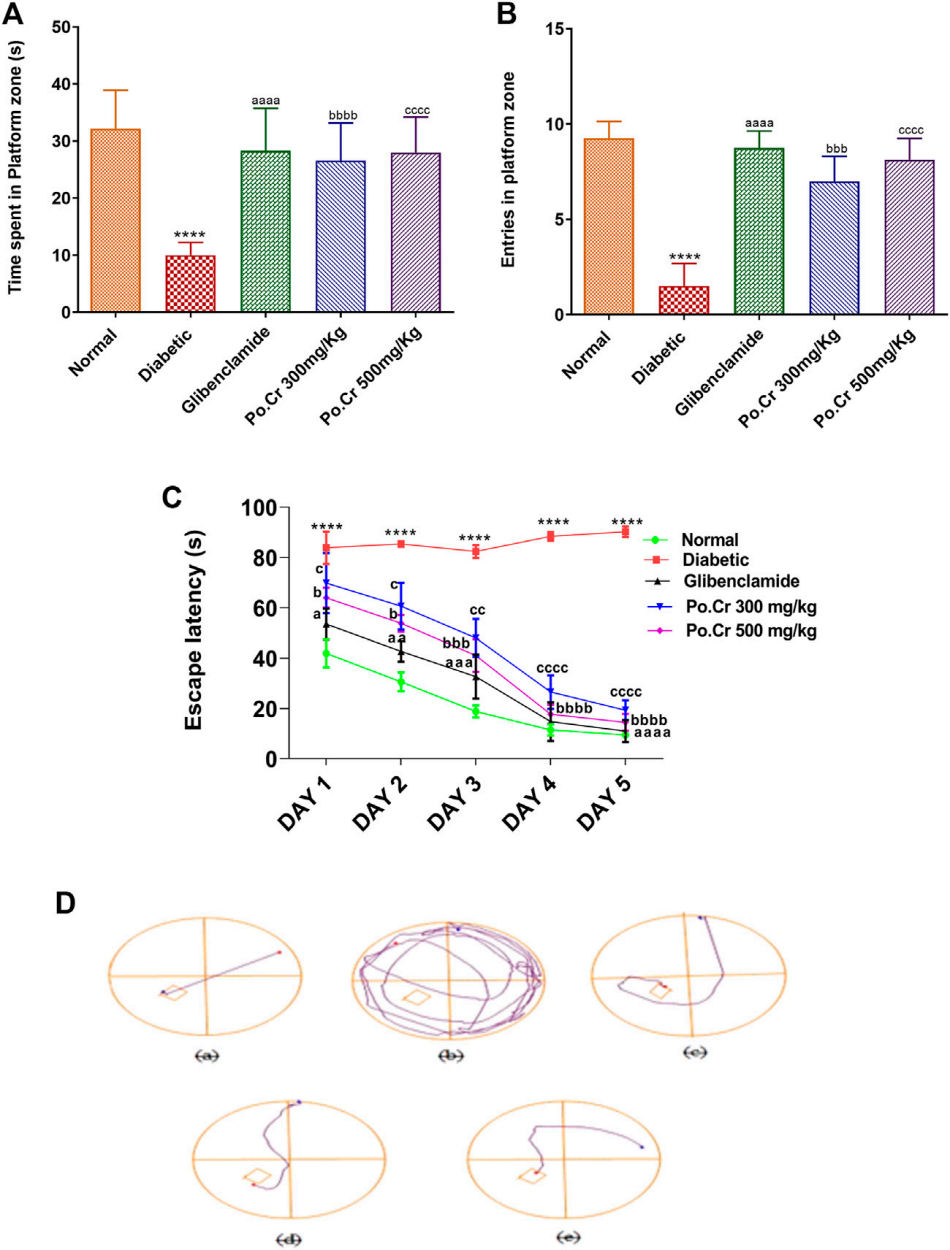
Representation of the effects of crude methanolic extract of *Plumeria obtusa* on the performance of diabetic rats in the Morris water maze test. **(A)** Time spent in platform (SW) zone, **(B)** entries into platform (SW) zone, **(C)** escape latency, and **(D)** swim paths taken by rats to the hidden platform in the SW zone. Group II (diabetic control) was compared to Group I (normal control), whereas all the treated groups (Groups III [glibenclamide] to V [Po.Cr]) were compared with Group II (diabetic control). ANOVA (two-way) and the multiple comparison Dunnett’s test were applied, and the data values are mean ± SEM. *p < 0.05 indicates comparison of diabetic control (Group II) to normal control (Group I), whereas ^a^ *p* < 0.05, ^b^ *p* < 0.05, and ^c^ *p* < 0.05 indicate comparison of glibenclamide (Group III); Po.Cr, 300 mg/kg (Group IV); and Po.Cr, 500 mg/kg (Group V) *versus* the diabetic control group, respectively.

The Morris water maze test was performed to explore the effects of Po.Cr on learning and memory in diabetic rats. Our results indicated that escape latency and distance traveled by normal control rats to reach the hidden platform was reduced, whereas the number of entries into the platform zone (SW zone) increased as they were trained over 4 days of testing. In contrast, diabetic rats exhibited thigmotaxic behavior and reduced capacity for task execution as escape latency and distance traveled to reach the platform increased, along with decreased numbers of entries into the SW zone compared to the control group (Figures 2A–D). The results also indicated that treatment of diabetic rats with Po.Cr (300 or 500 mg/kg) led to a marked increase in performance (*p* < 0.001) as demonstrated by more rapid location of the platform in comparison to diabetic rats that did not receive Po.Cr. The probe day results showed that time spent in the platform zone (Figure 2A) and the number of entries into the platform zone (Figure 2B) were decreased in diabetic rats, and the total distance traveled to reach the platform was increased in comparison to the control group. Rats treated with Po.Cr at either dose, however, presented a significant (*p* < 0.001) increase in number of entries and time spent in the target quadrant and a significant (*p* < 0.001) reduction in distance traveled to reach the target quadrant in comparison to diabetic rats that did not receive Po.Cr. Overall, these data suggest that *Plumeria obtusa* L. can attenuate the learning and cognitive deficits observed in diabetic rats (Figures 2A–D).

#### 3.3.2 Behavioral tests for anxiety

##### 3.3.2.1 Open field test

**FIGURE 3:**
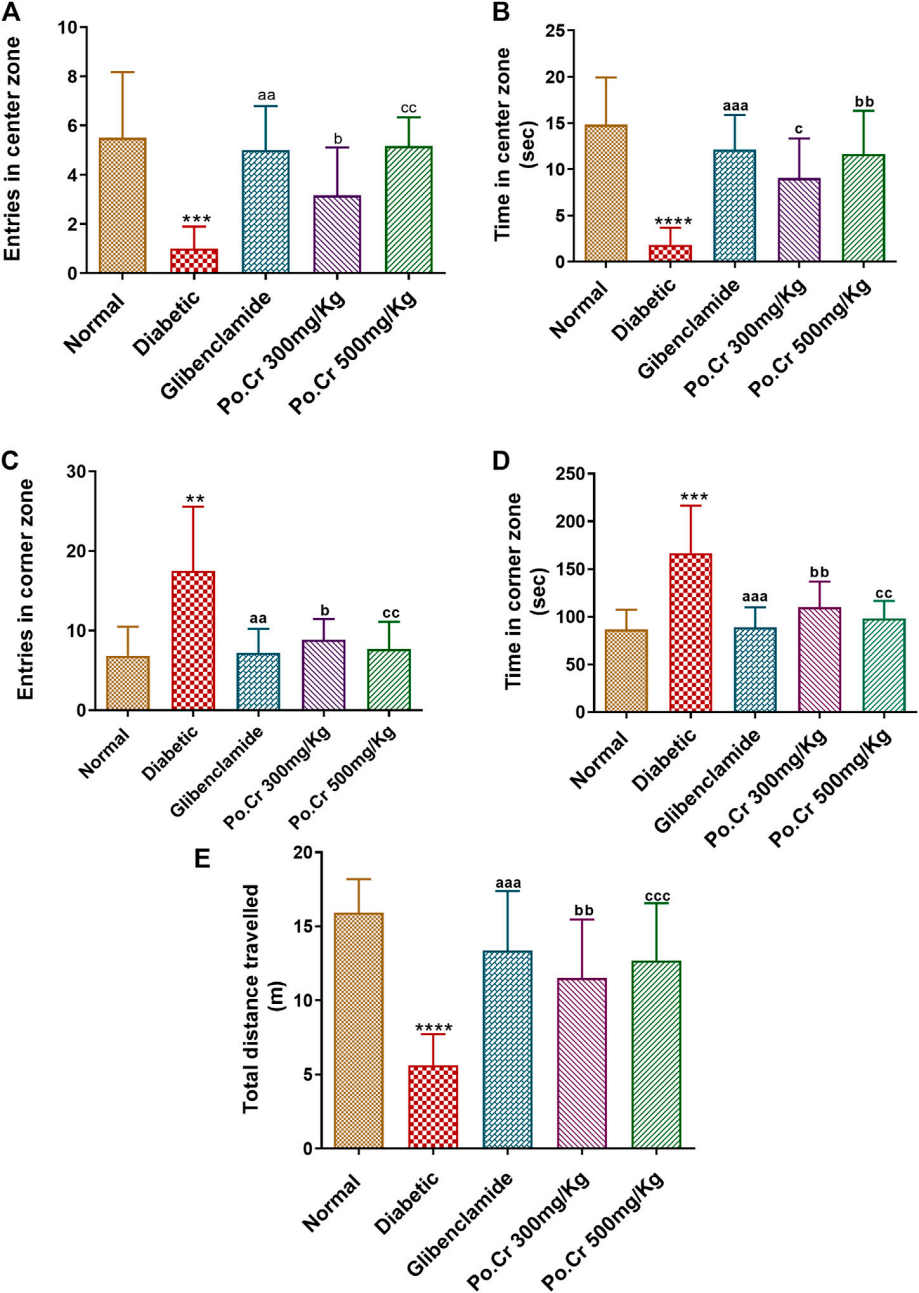
Anxiolytic capacity of aqueous extract of *Plumeria obtusa* at doses of 300 and 500 mg/kg assessed using the open field test. **(A)** Entries into center zone. **(B)** Time spent in center zone. **(C)** Entries into corner zone. **(D)** Time spent in corner zone. **(E)** Total distance travelled. Group II (diabetic control) was compared to Group I (normal control), whereas all the treated groups (Groups III [glibenclamide] to V [Po.Cr]) were compared with Group II (diabetic control). ANOVA (two-way) and the multiple comparison Dunnett’s test were applied, and the data values are mean ± SEM. *p < 0.05 indicates comparison of the diabetic control (Group II) with the normal control (Group I), whereas ^a^ *p* < 0.05, ^b^ *p* < 0.05, and ^c^ *p* < 0.05 indicate comparison of glibenclamide (Group III); Po.Cr, 300 mg/kg (Group IV); and Po.Cr, 500 mg/kg (Group V) *versus* the diabetic control group, respectively.

On day 25 of the experiment, animals were subjected to OFT an hour after administration of Po.Cr at one of the two doses and treatment with diazepam as a standard single dose. A significant difference was detected between groups in the number of entries into the center square (Figure 3A) and time spent in the center square (Figure 3B). There was a higher mean number of entries and greater time spent in corner squares in the diabetic group (Group II) compared to the normal (non-diabetic) group and the glibenclamide and Po.Cr treated groups (Figures 3C, D). After treatment with Po.Cr at either dose, there was a significant increase (*p* < 0.001) in total distance traveled (Figure 3E), number of entries, and time spent in the central zone of the open field apparatus, and a decrease in the number of entries and time spent in the corner zones in comparison to the diabetic control group (Group II) (*p* < 0.001). Outcomes were comparable to that of the standard and shown in Figures 3A–E).

##### 3.3.2.2 Elevated plus maze

**FIGURE 4:**
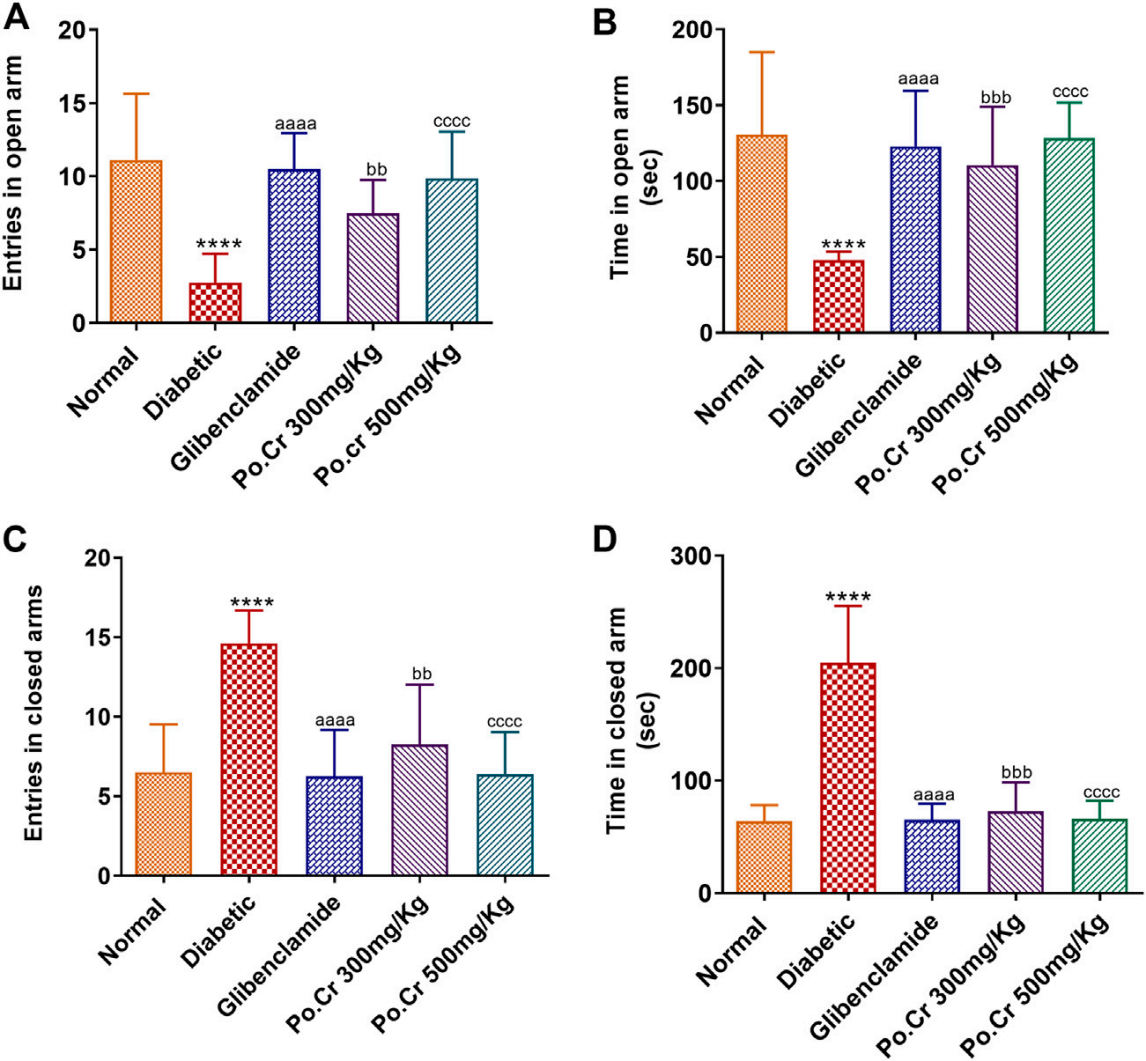
Anxiolytic effects of Po.Cr in the elevated plus maze (EPM) test of alloxan-induced diabetic rats. **(A)** Entries into open arm. **(B)** Time spent in open arm. **(C)** Entries into closed arm. **(D)** Time spent in closed arm. Group II (diabetic control) was compared to Group I (normal control), whereas all the treated groups (Groups III [glibenclamide] to V [Po.Cr]) were compared with Group II (diabetic control). ANOVA (two-way) and the multiple comparison Dunnett’s test were applied, and the data values are mean ± SEM. *p < 0.05 indicates comparison of diabetic control (Group II) to normal control (Group I), whereas ^a^ *p* < 0.05, ^b^ *p* < 0.05, and ^c^ *p* < 0.05 indicates comparison of glibenclamide (Group III); Po.Cr, 300 mg/kg (Group IV); and Po.Cr, 500 mg/kg (Group V) *versus* the diabetic control group, respectively.

Experimental animals treated with Po.Cr at either dose were exposed to the field of elevated-plus maze. Results showed that alloxan-induced diabetic rats demonstrated anxiety-like behavior, including significantly decreased number of entries and time spent in open arms of the apparatus and increased number of entries and time spent in closed arms compared to normal control rats. Moreover, the results indicated that rats treated with Po.Cr at either dose were less anxious, as they made fewer entries and spent shorter periods of time in closed arms of the maze (*p* < 0.01–p<0.001) compared to diabetic control rats (Figures 4A–D).

##### 3.3.2.3 Light and dark aversion test

**FIGURE 5:**
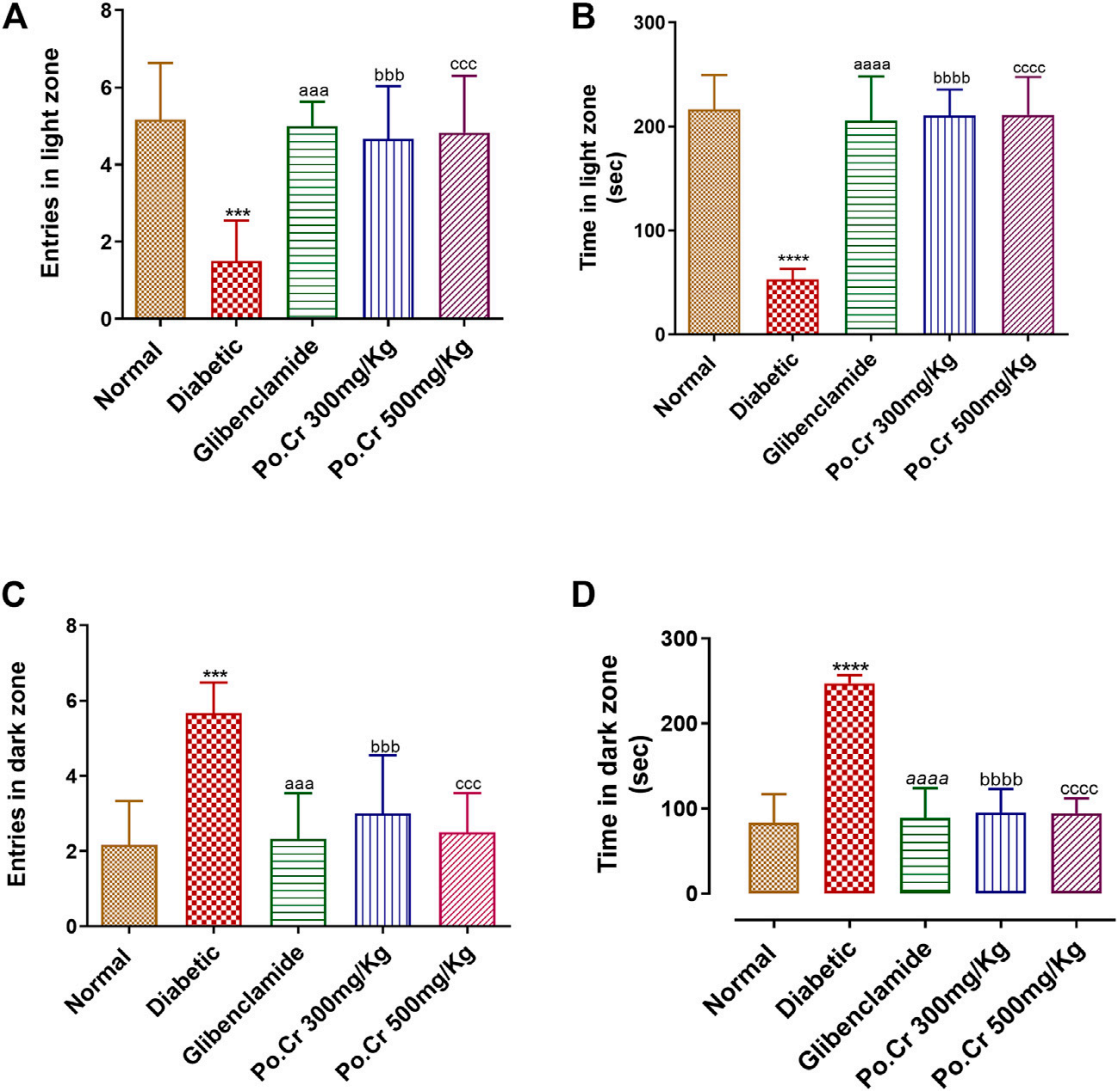
Anxiolytic effects of Po.Cr on alloxan-induced diabetic rats in the light and dark (L/D) test. **(A)** Entries into the light zone. **(B)** Time spent in the light zone. **(C)** Entries into the dark zone. **(D)** Time spent in the dark zone. Group II (diabetic control) was compared to Group I (normal control), whereas all the treated groups (Groups III [glibenclamide] to V [Po.Cr]) were compared with Group II (diabetic control). ANOVA (two-way) and the multiple comparison Dunnett’s test were applied, and the data values are mean ± SEM. *p < 0.05 indicates comparison of the diabetic control (Group II) to the normal control (Group I), whereas ^a^ *p* < 0.05, ^b^ *p* < 0.05, and ^c^ *p* < 0.05 indicates comparison of glibenclamide (Group III); Po.Cr, 300 mg/kg (Group IV); and Po.Cr, 500 mg/kg (Group V) *versus* the diabetic control group, respectively.

Experimental animals of each group were subjected to the L/D aversion test to further explore anxiety-like behavior. The results revealed that diabetic rats are more anxious compared to normal rats, as demonstrated by reduction in time spent and number of entries into the light chamber and by increased time spent and number of entries into the dark chamber of the L/D box. Furthermore, Po.Cr at either dose decreased anxiety in diabetic rats as demonstrated by significantly increased time spent and number of entries in the light chamber and decreased time spent and number of entries in the dark chamber compared to diabetic rats without Po.Cr treatment (*p* < 0.001), as shown in Figure 5.

#### 3.3.3 Behavioral test for depression

**FIGURE 6:**
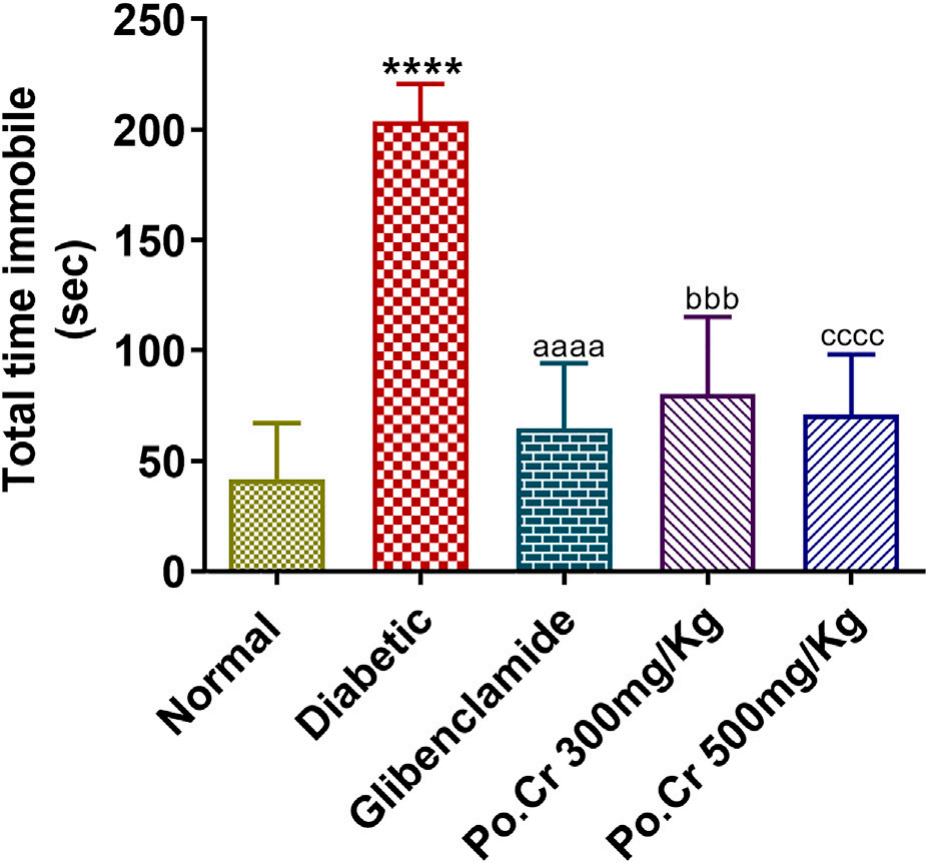
Anti-depressant effects of Po.Cr in the forced swim test (FST) of alloxan-induced diabetic rats, showing total time immobile (in sec) for the different groups. Group II (diabetic control) was compared to Group I (normal control), whereas all the treated groups (Groups III [glibenclamide] to V [Po.Cr]) were compared with Group II (diabetic control). ANOVA (two-way) and the multiple comparison Dunnett’s test were applied, and the data values are mean ± SEM. *p < 0.05 indicates comparison of the diabetic control (Group II) to the normal control (Group I), while ^a^ *p* < 0.05, ^b^ *p* < 0.05, and ^c^ *p* < 0.05 indicates comparison of glibenclamide (Group III); Po.Cr, 300 mg/kg (Group IV); and Po.Cr, 500 mg/kg (Group V) *versus* the diabetic control group, respectively.

Animals of all experimental groups were forced to swim to allow investigation of the anti-depressant effects of Po.Cr and of fluoxetine as standard treatment. The results indicated that diabetic rats showed increased duration of immobility and a decreased mobility period compared to normal control rats. Treatment with Po.Cr at either dose significantly reduced the immobility period and increased the mobility time compared to diabetic control rats (*p* < 0.001) (Figure 6).

### 3.4 Po.Cr demonstrates strong antioxidant potential

**FIGURE 7:**
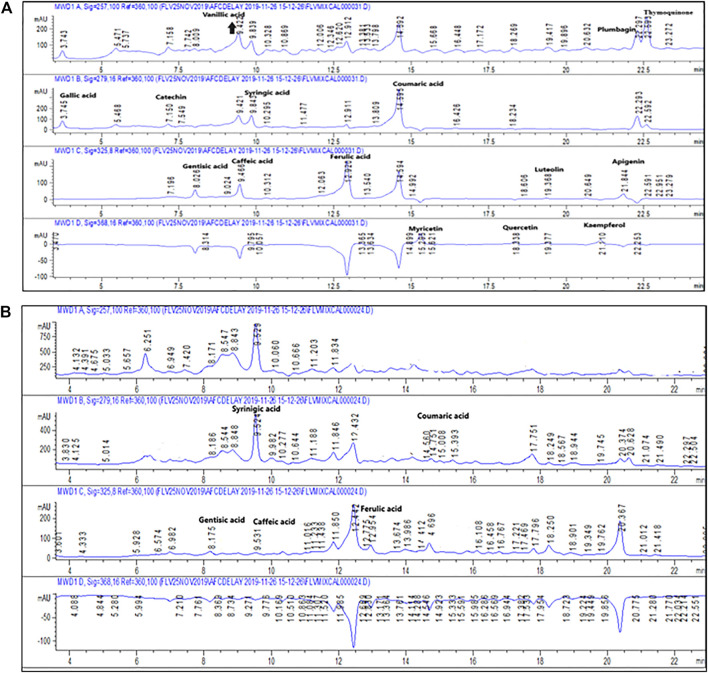
**(A)** HPLC chromatogram of the standard sample. **(B)** HPLC chromatogram of methanolic extract of *Plumeria obtusa* L.

In DM, there is disturbance of the redox equilibrium and more and more free radicals are generated (Matough et al., 2012). Neurodegenerative effects of ROS are often found to be responsible for Alzheimer disease symptoms. Several experiments were performed to study the impact of Po.Cr on free radical scavenging. Phytochemical assays were carried out to identify active constituents of Po.Cr that have antioxidant potential. The results indicated the TPC and TFC in Po.Cr were 53.11 ± 1.90 gallic acid equivalents (GAE)/mg extract and 38.19 ± 0.98 quercetin equivalents (QE)/mg extract, respectively. HPLC-DAD analysis demonstrated the presence of syringic acid, coumaric acid, emodin, gentisic acid, and caffeic acid in the aqueous-methanolic extract of *Plumeria obtusa*, as shown in Table 3; Figures 7A, B. Po.Cr exhibited excellent antioxidant and free radical scavenging properties (Table 4). Overall, these results indicate the presence of several antioxidant compounds in the methanolic extract of Po.Cr (HPLC analysis) and the antioxidant and anti-diabetic potential of this traditional medicine *via* the improvement of the metabolic profile and neuropsychiatric symptoms in diabetic rats.

**TABLE 3:** Compounds identified in methanolic extract of *Plumeria obtusa* L. using HPLC-DAD.

Compound	Groups	Quantity
Syringic acid	Phenols	1.14 (μg/mg DW)
Coumaric acid	Hydroxycinnamic acid	0.21 (μg/mg DW)
Emodin	Trihydroxyanthraquinone	0.77 (μg/mg DW)
Gentisic acid	Dihydroxybenzoic acid	1.25 (μg/mg DW)
Caffeic acid	Hydroxycinnamic acid	0.33 (μg/mg DW)
Ferulic acid	Hydroxycinnamic acid	0.62 (μg/mg DW)

The calculated IC_50_ values of different antioxidant activities are given in Table 4.

**TABLE 4:** Assessment of antioxidant markers.

Antioxidant marker	IC_50_	Unit
DPPH radical-scavenging activity	16.56 ± 1.43	μg/ml
Total antioxidant capacity	226.21 ± 1.57	Ascorbic acid equivalents (AAE)/mg extract
Total reducing power	390.33 ± 1.35	Ascorbic acid equivalents (AAE)/mg extract

## 4 Discussion

**FIGURE 8:**
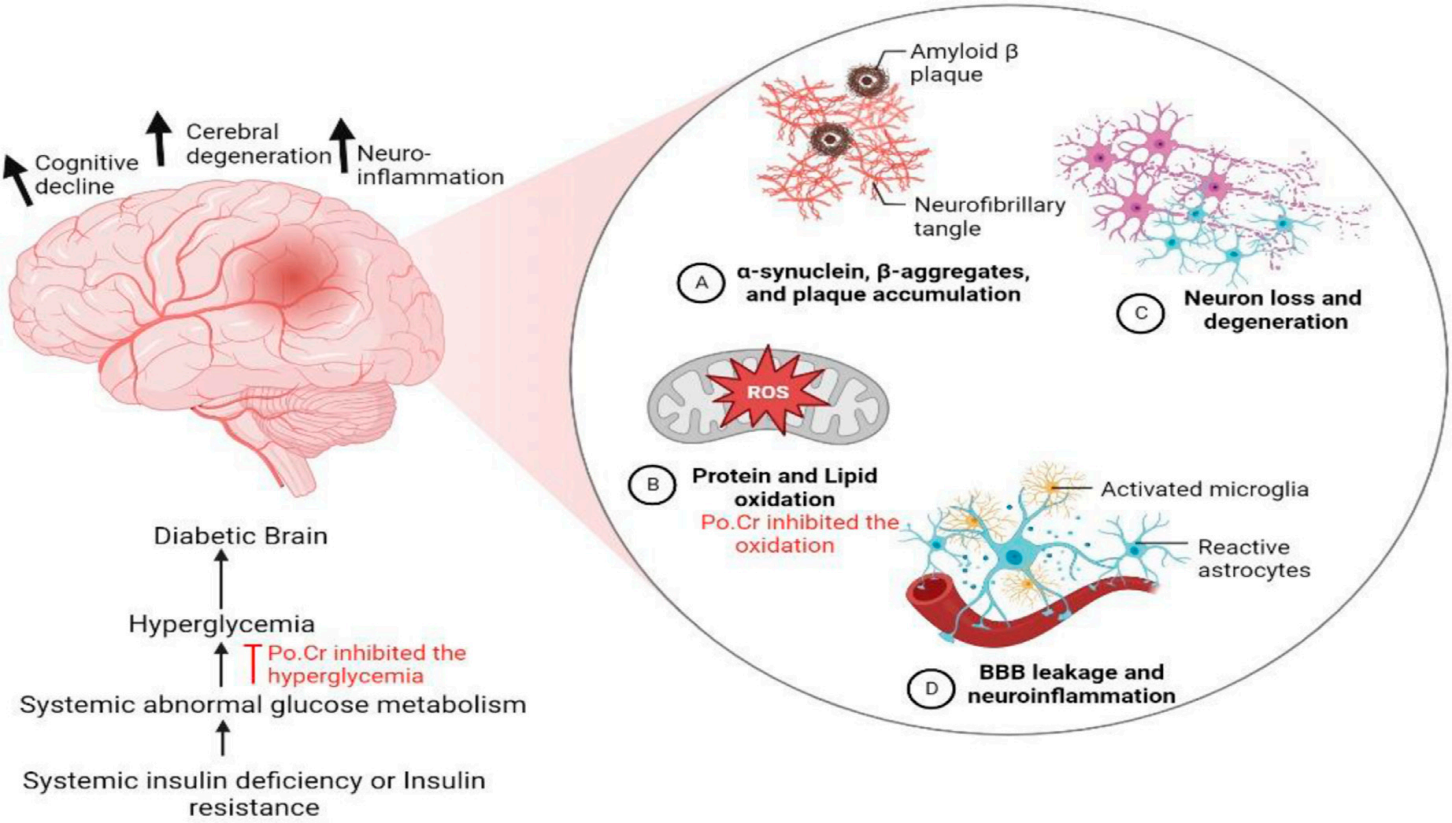
Illustration explaining the possible mechanism of development of cognitive impairment and dysfunction associated with diabetes mellitus. The long-term administration of crude extract of *Plumeria obtusa* L. not only reduces blood glucose level but also reduces oxidative stress and therefore prevents diabetes-associated neuroinflammation and cognitive dysfunction.

The prevalence of metabolic and neurodegenerative complications is increasing with time in the developed world. Diabetes is the most common metabolic disorder affecting the population worldwide and is associated with numerous microvascular and macrovascular complications. Both type-1 and type-2 DM are found to have close association with cognitive dysfunction. Early cognitive deficits in learning and memory and in mental flexibility and speed might be associated with diabetes as depicted in Figure 8 (Sims-Robinson et al., 2010). Various available anti-diabetic medicines were found to exert limited control over the glycemic index and associated cognitive complications, which stimulated researchers to search for novel therapeutics to address this critical health challenge (Mechchate et al., 2021). Natural resources have gained attention among researchers worldwide for use in the development of novel therapeutics due to their attractive safety profile and economic benefits. Our study revealed that crude methanolic extract of *Plumeria obtusa* L. (Po.Cr) significantly attenuates diabetes and associated Alzheimer-like symptoms in an alloxan-induced diabetic rat model. Phytochemical analysis using high-performance liquid chromatography confirmed the presence of flavonoids, phenols, and phenolic acids, including syringic acid, coumaric acid, ferulic acid, caffeic acid, and gentisic acid in *Plumeria obtusa* L extract. Furthermore, antioxidant and free radical scavenging activity of Po.Cr was confirmed, which may be due to the presence of flavonoids, phenols, and phenolic acids.

Single dose alloxan monohydrate resulted in increased blood glucose levels and decreased body weight in rats for about 45 days. These parameters presented effective establishment of diabetes in rats, as similar findings have been reported previously (Yin et al., 2018). Long-term administration of Po.Cr significantly controlled blood glucose level and improved weight compared to diabetic rats that did not receive Po.Cr. The flavonoids in Po.Cr (Table 3) might exert a hypoglycemic effect through stimulation of insulin secretion, as demonstrated in previous studies (Baharvand-Ahmadi et al., 2016). Flavonoids and phenolic compounds are found to have several benefits against many disorders, including diabetes (Sarian et al., 2017; Memariani et al., 2021), by targeting different pathways and affecting β-cell proliferation, as well as insulin signaling and secretion (Graf et al., 2005).

The diabetes-like metabolic disorders are associated with dyslipidemia due to elevated ROS and related oxidative stress (Samarghandian et al., 2013). An accumulation of triglycerides and LDL, and reduced HDL levels were found in alloxan-induced diabetic female rats (Júnior et al., 2017), which might be due to reduced utilization of glucose and additional disposal of fats from adipose tissues (Draganescu et al., 2021). Our study showed that long-term administration of Po.Cr reduces the hyperlipidemia associated with diabetes, which suggests the presence of phenols and flavonoids that might enhance insulin release from pancreatic β-cells, as well as decrease LDL oxidation (Fuhrman and Aviram, 2001; Hossain et al., 2011). Furthermore, phenols attenuate oxidative stress and inflammatory mediators (including *Nf*-KB), and reduce the production of eicosanoid derivatives by inhibiting the arachidonic cascade (Feldman et al., 2021; Aleem et al., 2022). Furthermore, previous experiments suggested that alloxan-induced diabetes affects multiple organ systems, including the liver (Lucchesi et al., 2015). Hepato-cellular injury was indicated by increased levels of ALT enzymes in this study, which might have been due to toxic effects of alloxan and/or the diabetic state of the rats (Aleissa et al., 2020). Administration of Po.Cr reduced the ALT levels, which may have been mediated by flavonoids in the extract. Flavonoids have been shown to reduce inflammation and oxidative stress in hepatic cells and modulate pathways of insulin signaling and liver gluconeogenesis (Yin et al., 2018; Kang et al., 2020), and are potential contributors to the hepatoprotective effects observed in Po.Cr-treated diabetic rats.

Both types of diabetes result in increased production of ROS (Matough et al., 2012), which is a contributing factor in diabetic neuropathy. Alloxan induces diabetes through intracellular generation of ROS, with subsequent increases in cytosolic calcium level and thus oxidative pressure through reduction of endogenous anti-oxidation mechanisms (Ceretta et al., 2012b) following the suppression of insulin release and synthesis (Rohilla and Ali, 2012). Some of the anti-diabetic potential of Po.Cr in the alloxan-induced diabetic rat model may be due to the antioxidant potential of phenols and flavonoids contained in Po.Cr extract that combat the oxidative stress, mediated by alloxan, that affects pancreatic β-cells.

The metabolic signaling *via* glucose and insulin are important phenomenon for healthy activity of brain (Sims-Robinson et al., 2010). Therefore, diabetes has been associated with cognitive deficit and psychiatric comorbidities (Raffield et al., 2016). Dementia and cognitive impairment are common complications of DM, and elderly patients with DM are at higher risk of developing Alzheimer disease due to serious neuronal damage (Jiang et al., 2012; Behl et al., 2021). The prospective mechanisms for this incorporate direct impacts of hypo or hyperglycemia and hypo or hyperinsulinemia and indirect impacts include increased intracellular calcium levels, mitochondrial dysfunction, oxidative stress, and neurochemical changes that cause cerebrovascular modification (Sims-Robinson et al., 2010; Li et al., 2019; Xu et al., 2021; Song and Wu, 2022). The Morris water maze (MWM) test is one of the most widely used models for the assessment of memory and learning. The results from the MWM test in our study indicate improved memory in Po.Cr-treated diabetic rats compared to untreated diabetic rats. Phenols and flavonoids have been reported to have neuroprotective effects by controlling neuroinflammation, reducing oxidative stress and neuronal dysfunction, and improving neuronal differentiation in the hippocampus (Vauzour, 2012; Hussain et al., 2018). Thus, the antioxidant capacity of Po.Cr might be due to presence of phenols and flavonoids that regulate the levels of antioxidant enzymes and attenuate neuronal damage in rat brains (Dogra, 2016; Singh et al., 2020; Ul Hassan et al., 2021).

Several previous studies have documented an association between diabetes and psychiatric disorders, such as anxiety and depression, that is potentially due to a disturbance in levels and functions of some neurotransmitters, including those that are serotonergic, GABAergic, dopaminergic, or noradrenergic, caused by increased glucose level (Shpakov et al., 2011). Patients with DM are 14.3 times more likely to develop comorbid depression and expresses poor glycemic control and poor adherence to diet and medicine (Andreoulakis et al., 2012). Reagan (2012) reported that similar psychiatric problems were observed in diabetic animal models. In our study, exaggerated symptoms of anxiety-like behavior were noted in diabetic rats as they stayed longer in hidden and darker areas during the experiment and treatment with Po.Cr at either dose significantly attenuated the anxiety compared to untreated diabetic rats. The anxiolytic effects of Po.Cr might be due to the presence of flavonoids, phenols, and terpenoids in Po.Cr extract. It has been indicated in a previous report that flavonoids and phenols found in natural medicinal plants are known to augment GABA_A_ receptor neurotransmission in the brain and have additional antioxidant properties (Singh et al., 2012; Komaki et al., 2016; Muhasaparur Ganesan et al., 2021). Likewise, the Po.Cr treatment of diabetic rats attenuated depression-like symptoms, resulting in increased mobility in FST compared to untreated diabetic rats. Phenols and polyphenols may attenuate depression by regulating monoamine neurotransmitters in the brain (Li et al., 2020) and the antioxidant potential of flavonoids and phenols may alleviate depressive behavior by protecting the brain from oxidative stress and neuronal damage.

The results of our study suggest that long-term administration of crude methanolic extract of *Plumeria obtusa* L. not only attenuates hyperglycemia in alloxan-induced diabetic rats, but also improves associated metabolic disorders, Alzheimer-like symptoms, and psychiatric disorders, potentially due to the presence of phytochemical constituents with strong antioxidant capacity.

## 5 Conclusion

The findings of our study revealed the presence of flavonoids and phenolic compounds in crude methanolic extract of *Plumeria obtusa* L. Po.Cr attenuates diabetes, and controls body weight, liver function enzyme levels, and lipid profile parameters in an alloxan-induced diabetic rat model. Moreover, Po.Cr improved diabetes-associated cognitive impairment and psychiatric disorders in diabetic rats, which may be due to its antioxidant capacity and prevention of neuronal damage resulting from oxidative stress. These data demonstrate the importance of further study of the potential of Po.Cr in providing protection against the development of Alzheimer disease in patients with diabetes. This study provides scientific evidence that supports the traditional uses of this plant, yet further investigation is required to clarify the mechanisms responsible for the beneficial effects of *Plumeria obtusa* L. in the treatment of diabetes.

## Data Availability

The original contributions presented in the study are included in the article/Supplementary Material; further inquiries can be directed to the corresponding authors.

## Abbreviations

AAE/DW, the number of mg equivalents of ascorbic acid per gram of dry plant weight; DM, diabetes mellitus; DPPH, 2,2-diphenyl-1-picrylhydrazyl; EPM, elevated plus maze; FST, forced swim test; HPLC, high performance liquid chromatography; MWM, Morris water maze; Po.Cr, crude methanolic extract of *Plumeria obtusa* L.; ROS, reactive oxygen species; TFC, total flavonoid content; TPC; total phenolic content.
